# Sutural Cataract Associated With Pupillary Membrane: A Case Report of an Unusual Relation

**DOI:** 10.7759/cureus.69027

**Published:** 2024-09-09

**Authors:** Jorge S Saucedo Rizo, Omar A Hernandez Rodriguez

**Affiliations:** 1 Department of Ophthalmology, Hospital Central Sur de Alta Especialidad, Ciudad de México, MEX

**Keywords:** cataract, congenital cataract, genetics, ophthalmology, pupillary membrane, sutural cataract

## Abstract

Sutural cataracts and pupillary membranes are congenital abnormalities, the former being an atypical type of congenital cataract resulting from the opacification of the lens sutures, and the latter developing from the remnants of the tunica vasculosa lentis. While genetic mutations related to sutural cataracts are becoming increasingly known and pupillary membranes are sometimes related to polar cataracts, no genetic studies have been conducted to link these two alterations. We present an unusual case of a woman with an unnoticed congenital sutural cataract and a pupillary membrane in opposite eyes.

## Introduction

During embryological stages, epithelial cells forming the anterior and equatorial aspects of the early crystalline lens divide and elongate to meet each other at the anterior and posterior poles of the lens, thus forming fiber structures. These fibers, known as sutures, become larger and more numerous as the lens grows remaining mostly transparent throughout life [1].

Sutural cataract is an uncommon opacity of the fetal lens sutures, often forming a “Y”-like shape [2]. In contrast, a pupillary membrane is a remnant of an embryonic capillary network of the lens, and its persistence is associated with certain types of polar cataracts. Though mutations in seven genes, as well as other syndromic diseases, such as Axenfield Rieger syndrome and Nance Hooran syndrome, are linked to sutural cataracts, no specific genetic associations have been identified between sutural cataracts and pupillary membranes [1-2]. The visual prognosis is usually good in both pathologies, although cataract surgery could be beneficial in sutural cataracts with low visual acuity. This case report presents an unusual instance in which a pupillary membrane and a sutural cataract coexist, suggesting a probable genetic relationship between these two congenital anomalies.

## Case presentation

**Figure 1:**
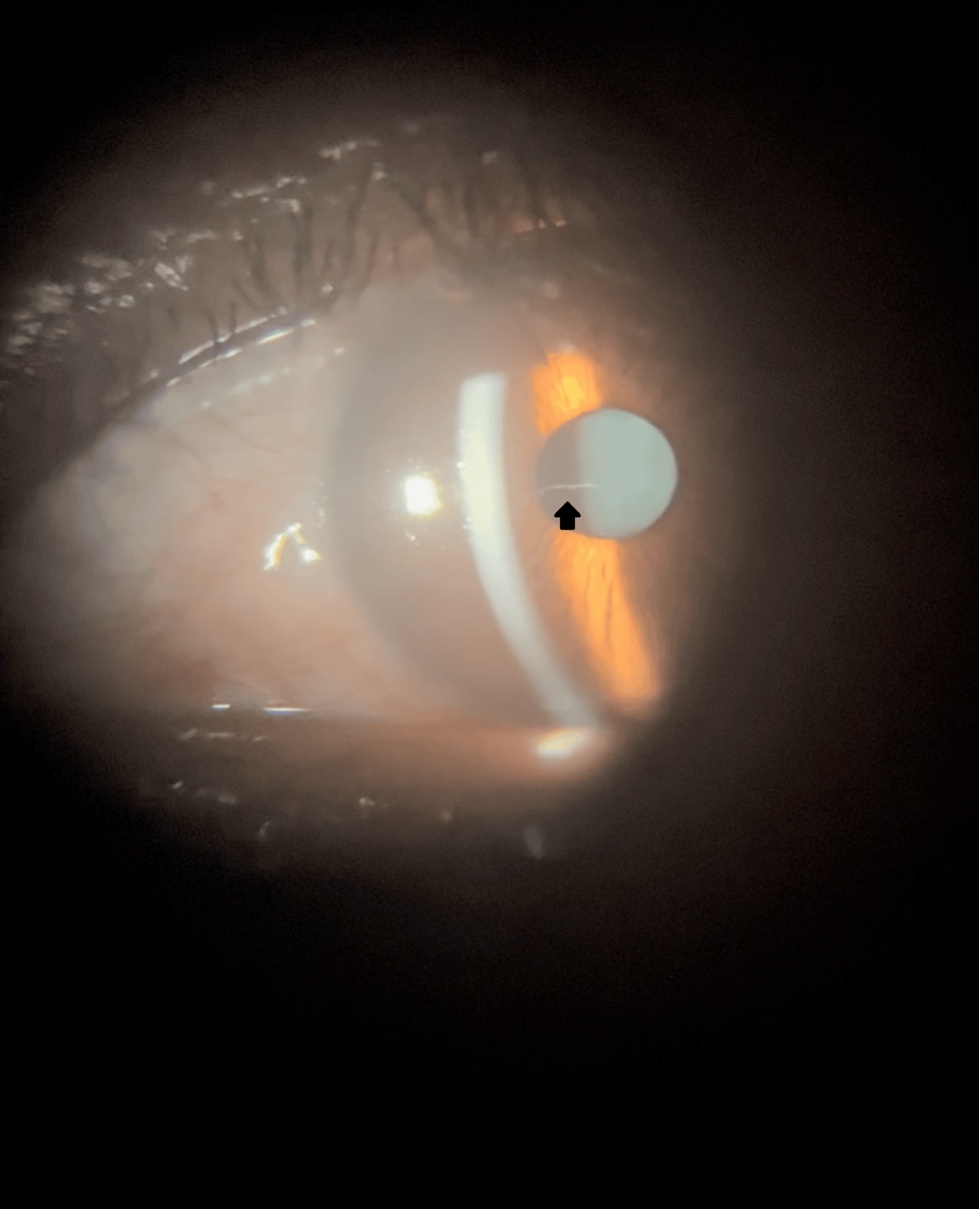
Clinical picture of the right eye showing a translucent fibrous structure in front of the pupil, characteristics of a pupillary membrane

**Figure 2:**
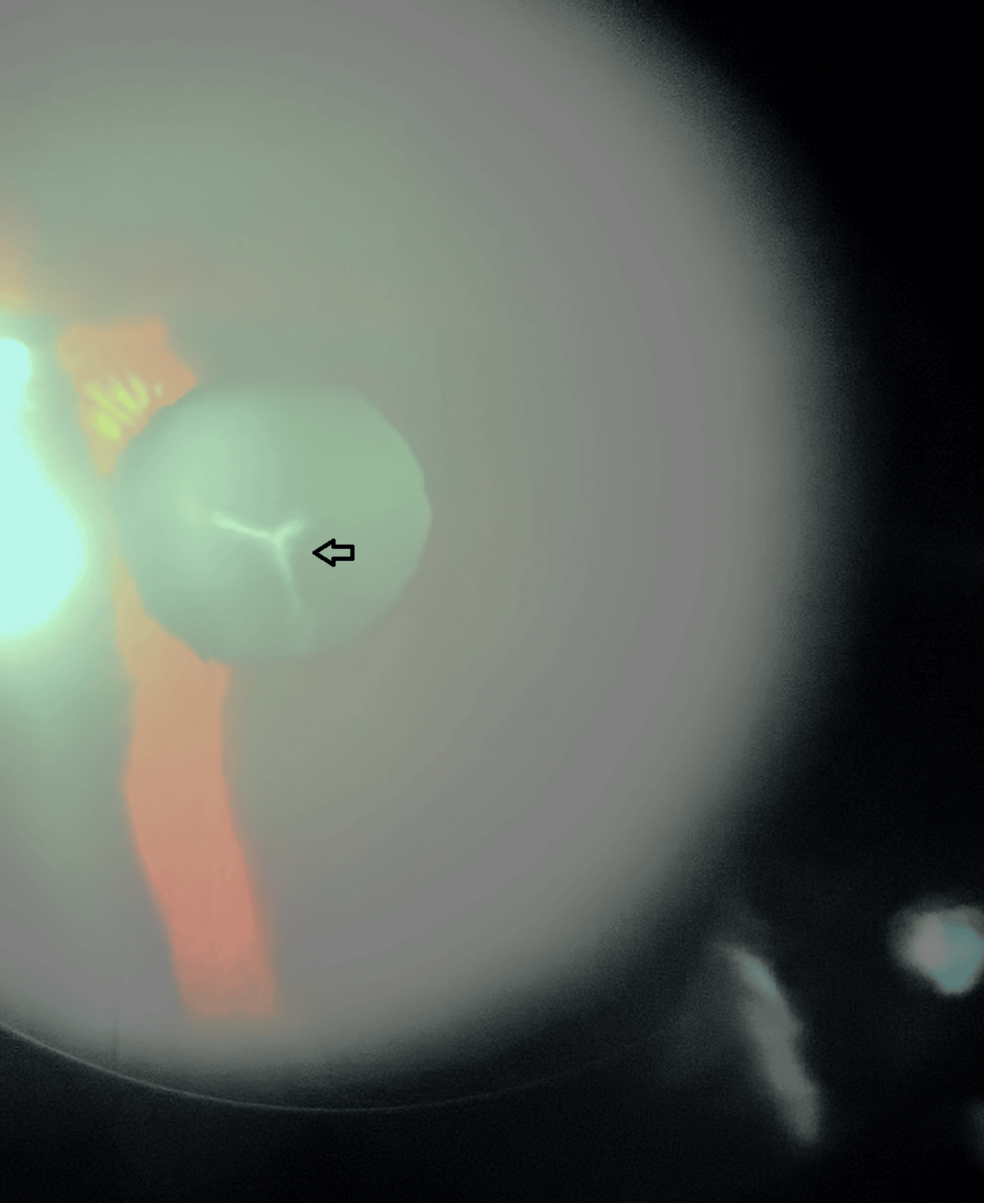
Clinical picture of the left eye depicting a "Y"-like opacification of the crystalline lens

We present the case of a 45-year-old woman who attended a routine ophthalmologic consultation. Although there was no history of clinically relevant pathology, she was concerned about an “opacification” that was seen by an optometrist during her last refraction. Her best corrected visual acuity was 20/20 in the Snellen chart (LogMAR 0) for the right eye and 20/30 (LogMAR 0.2) in the left eye. Biomicroscopy revealed clear corneas in both eyes; however, a small pupillary membrane was present in the right eye (Figure 1). Gonioscopy showed no signs of angle closure or anterior synechiae and the lens was clear. Additionally, a “Y”-shaped lens opacity was noted in the left eye (Figure 2). Fundus examination revealed no further abnormalities. Due to the good visual acuity, close observation of the cataract evolution was proposed to the patient.

## Discussion

Although congenital cataracts are a frequent cause of blindness in children according to the World Health Organization (WHO), sutural cataracts are uncommon. One study aimed at analyzing the difference between congenital cataracts in 83 children found that a sutural cataract was present in less than 5% of patients [3].

Currently, sutural cataracts have been associated with multiple genetic mutations. Sequential changes have been identified along seven genes, namely, BFSP2, CRYBBA, CRYBB2, GJA8, FTL, CRYGA, and CRYBA1/A3, the latter having at least four mutations identified [4]. Likewise, mutations in regions D17S798 and D17S799 of the 17q24 chromosome have been linked to autosomal dominant sutural cataracts [5]. Moreover, certain genetic syndromes, such as cranio-lenticulo-sutural dysplasia syndrome and Nance Horan syndrome, have been associated with sutural cataracts [6].

Sutural cataracts rarely cause any symptoms, thus, specific treatment is often unnecessary. However, blurred vision, glare, nyctalopia, or dyschromatopsia might be present; in that case, phacoemulsification surgery is the gold standard treatment [7].

On the other hand, pupillary membrane is a congenital ocular anomaly in which remnants of the tunica vasculosa lentis can be seen as fine to gross translucent strands along the pupil or pupillary margin [8].

Although most cases are sporadic, there have been familial occurrences reported in literature suggesting a potential genetic component, though, no gene has been conclusively linked [9]. Additionally, some ocular abnormalities are known to be rarely related to persistent pupillary membranes, for instance, coloboma, corneal opacities, anterior segment abnormalities, strabismus, as well as anterior and polar cataracts [10].

This case depicts an unusual occurrence of a unilateral persistent pupillary membrane and a unilateral sutural cataract in the opposite eye. Even though it is not described in the current literature, a genetic association between these two congenital anomalies might exist. Further genetic studies are needed to confirm this hypothesis.

## Conclusions

This case highlights an unusual finding of a unilateral sutural cataract and a pupillary membrane in the opposite eye. While no specific genetic association has been confirmed between these two congenital anomalies, the presence of such conditions in the same patient suggests the possibility of a shared genetic or developmental origin. Further genetic research, including examining the patient's family members (siblings, children, and parents), may provide insight into whether these conditions are part of a hereditary pattern or related to specific genetic mutations. Such studies could help confirm a potential genetic link and enhance understanding of these rare ocular anomalies.
